# Signatures of the Consolidated Response of Astrocytes to Ischemic Factors In Vitro

**DOI:** 10.3390/ijms21217952

**Published:** 2020-10-26

**Authors:** Elena V. Mitroshina, Mikhail I. Krivonosov, Dmitriy E. Burmistrov, Maria O. Savyuk, Tatiana A. Mishchenko, Mikhail V. Ivanchenko, Maria V. Vedunova

**Affiliations:** 1 Institute of Biology and Biomedicine, Lobachevsky State University of Nizhni Novgorod, 23 Prospekt Gagarina, 603950 Nizhny Novgorod, Russia; diman-burmistrov@yandex.ru (D.E.B.); mary.savyuk@bk.ru (M.O.S.); saharnova87@mail.ru (T.A.M.); 2 Institute of Information, Technology, Mathematics and Mechanics, Lobachevsky State University of Nizhni Novgorod, 23 Prospekt Gagarina, 603950 Nizhny Novgorod, Russia; mike_live@mail.ru (M.I.K.); ivanchenko.mv@gmail.com (M.V.I.)

**Keywords:** astrocytes, astrocytic networks, ischemia, connexin 43, calcium activity

## Abstract

Whether and under what conditions astrocytes can mount a collective network response has recently become one of the central questions in neurobiology. Here, we address this problem, investigating astrocytic reactions to different biochemical stimuli and ischemic-like conditions in vitro. Identifying an emergent astrocytic network is based on a novel mathematical approach that extracts calcium activity from time-lapse fluorescence imaging and estimates the connectivity of astrocytes. The developed algorithm represents the astrocytic network as an oriented graph in which the nodes correspond to separate astrocytes, and the edges indicate high dynamical correlations between astrocytic events. We demonstrate that ischemic-like conditions decrease network connectivity in primary cultures in vitro, although calcium events persist. Importantly, we found that stimulation under normal conditions with 10 µM ATP increases the number of long-range connections and the degree of corresponding correlations in calcium activity, apart from the frequency of calcium events. This result indicates that astrocytes can form a large functional network in response to certain stimuli. In the post-ischemic interval, the response to ATP stimulation is not manifested, which suggests a deep lesion in functional astrocytic networks. The blockade of Connexin 43 during ischemic modeling preserves the connectivity of astrocytes in the post-hypoxic period.

## 1. Introduction

For a long time, astrocytes have been thought to play an auxiliary role without generally affecting the higher functions of the central nervous system. However, recent experimental evidence has allowed for a more profound view on the functions of astrocytes and their role in adaptive processes in the CNS. Currently, there is no doubt that astrocytes are not simply passive transmitters of energy substrates and structural supports for neurons but also active participants in a large number of metabolic reactions [1]. Several studies have demonstrated the role of astrocytes in the pathological processes at sites of mechanical trauma and in the development of neurodegenerative diseases, such as Alzheimer’s disease and Parkinson’s disease [2,3,4].

While astrocytes are not electrically excitable cells, they are capable of producing and transmitting Ca2+ signals that can propagate between them, resulting in “calcium waves” [5]. It is known that astrocytes interact with neurons, mutually regulating the functional activity of each other [5]. At the same time, it remains unclear whether the astrocytes are able to respond to external stimuli and develop a consolidated response to stress independently on neurons. Investigating the collective dynamics of astrocytic calcium activity, particularly the coordinated activity, which would answer the long-standing problem of the existence of functional astrocytic networks, is highly anticipated.

In particular, a fundamental question is whether astrocytes are able to coordinate their activity over long distances. In other words, can a large-scale dynamic astrocytic network emerge in the absence of neurons? Another key question is whether a functional astrocytic network can be observed in the normal state or if its activity becomes manifested only after stimulation. Generally, the mechanisms that could possibly affect the strength of connections between cells in response to stress factors, thus reshaping the network, remain unknown. In this respect, elucidating the role of astrocytes in the neuroprotective function in the framework of functional astrocytic network reorganization would provide a novel perspective in understanding the processes of CNS adaptation.

Astrocytic interaction relies on gap junctions [6], which enable direct intercellular communication and transport of small molecules for maintaining homeostasis in the brain, glutamate, ATP, and Ca2+ [7]. Gap junctions are formed by two connexons (or hemichannels), and each of them consists of six connexin proteins. Overall, the brain expresses 11 connexins, among which connexin 43 (Cx43) is mostly attributed to astrocytes [6], affecting communication between them.

Understanding the influence of ischemia on the cellular network activity is also of paramount importance. While the resulting disturbance of neuronal activity has been well studied, much less is known regarding the alterations in functional consolidated astrocytic calcium activity. Arguably, elucidating the latter will pave the way to improving the therapy for ischemic stroke.

It is known that astrocytic gap junctions are normally open, while hemichannels formed by connexin 43 demonstrate a low probability of opening [8]. However, after an ischemic interval, the hemichannels become activated, which leads to the uptake of Na+ and Ca2+ into cells, accompanied by the release of ATP and other metabolites. This can cause changes in the calcium activity, an overload of Ca2+ up to toxic values, and osmotic imbalance up to cell death [9,10]. Additionally, ATP released from hemichannels can provoke neuroinflammation due to microglia and astrocyte activation [11]. Instructively, recent evidence demonstrates that hemichannel blockers, such as connexin 43 blocker GAP19, manifest neuroprotective effects in cerebral ischemia (reperfusion) [8].

Gap19 inhibits the opening of hemichannels without affecting gap junctions in astrocytes [12]. According to observations, selective inhibition of hemichannels Cx43 by Gap19 decreases the severity of a stroke and prevents the death of astrocytes in vivo and in vitro [13,14,15]. However, the effect of connexins 43 blockade on the consolidation of post-ischemic astrocytic calcium activity is currently unknown.

Primary astrocyte cultures could be considered as a model for studying the role of the consolidated astrocytic response because in such cultures, astrocyte–astrocyte interactions form without neuronal regulation, and the reactions of cells to various biochemical and physiological stimuli in isolation from neural activation can be observed.Accordingly, the study aimed to investigate the networks and related parameters of calcium activity in primary astrocytic cultures in a model of ischemic damage, as well as to estimate the neuroprotective effects of connexin 43 Gap19 blocker. For this purpose, we used the calcium event analysis tool for time-lapse fluorescence image recordings of astrocytic cultures [16] and developed an approach for reconstructing functional astrocytic networks based on inferred dynamic correlations. We demonstrate that model stress conditions and biochemical stimuli may produce a dramatic impact on the structure and statistics of these networks, suggesting the underlying changes in consolidated astrocytic activity and indicating the potential of the method for elucidating the effect of the other imposed conditions.

## 2. Results

### 2.1. ATP and Gap19 Effects on Functional Calcium Signaling in Astrocytes

The starting point of the study was estimating the level of consolidated calcium activity of primary astrocytic cultures under normal conditions. This relied on the novel method of computational analysis of time-lapse calcium imaging as described in the materials and methods.

**Figure 1:**
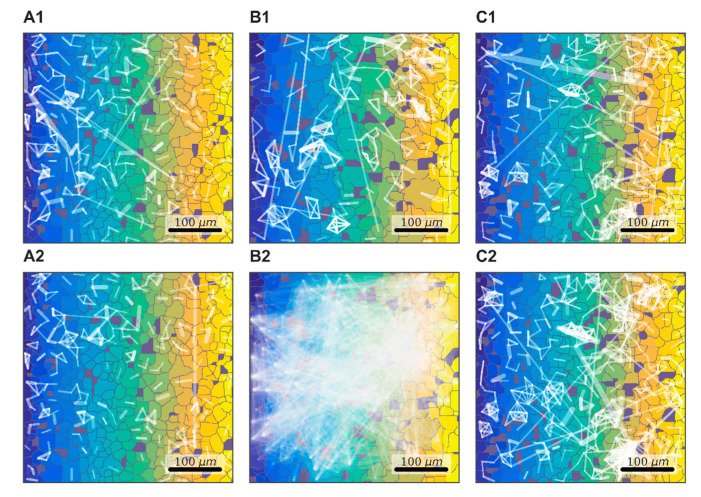
Representative examples of changes in correlation networks after ATP and connexin 43 blocker Gap19 application. (**A**) Control (PBS); (**B**) ATP (10 µM); (**C**) Gap19 (100 µM). (**1**)—before application; (**2**)—after application.

Astrocytes can easily exchange low-molecular-weight compounds (peptides up to 2 kDa, nucleotides, sugars) between adjacent cells through gap junctions that unite neighboring cells into a single functional conglomerate. However, such signals cannot propagate over long distances, as the speed of such passive transport is small and depends on the gradient of concentrations of low-molecular-weight substances in the cytoplasm of adjacent cells [17]. In this regard, an informative evaluation of functionally consolidated signals is given by remote in space and correlated in time calcium events in astrocytes. The correlation graph method showed a low-grade formation of functional networks between distant astrocytes in the “control” group of primary astrocyte cultures under normal conditions, characterized by a large number of disconnected subgraphs (Figure 1).

**Figure 2:**
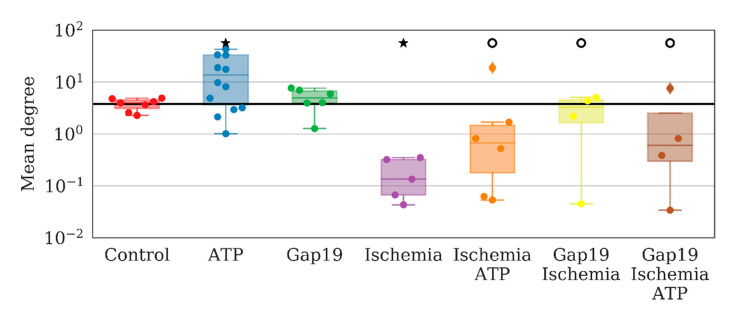
Average number of direct long-distance connections of a single astrocyte. ★—versus baseline, ○—versus “Ischemia”, *p* < 0.05, the Kolmogorov–Smirnov test.

To examine the role of one of the most common types of connexins (connexins 43) in the consolidated astrocyte response, we used the selective blocker Gap19 at a concentration of 100 µM/mL. The application of Gap19 for 40 min did not affect the number of direct long-distance connections per astrocyte under normal conditions, which was 4.94 [3.94; 6.68] (“control” 3.95 [3.10; 4.47]). To validate the ability of astrocytes to become activated and produce a coordinated response to biochemical stimuli, we used ATP (10 µM), which led to a significant 3.5-fold increase in the average number of direct long-distant connections of an astrocyte (13.63 [3.61; 33.23]) over 10 min after ATP application (Figure 2).

**Figure 3:**
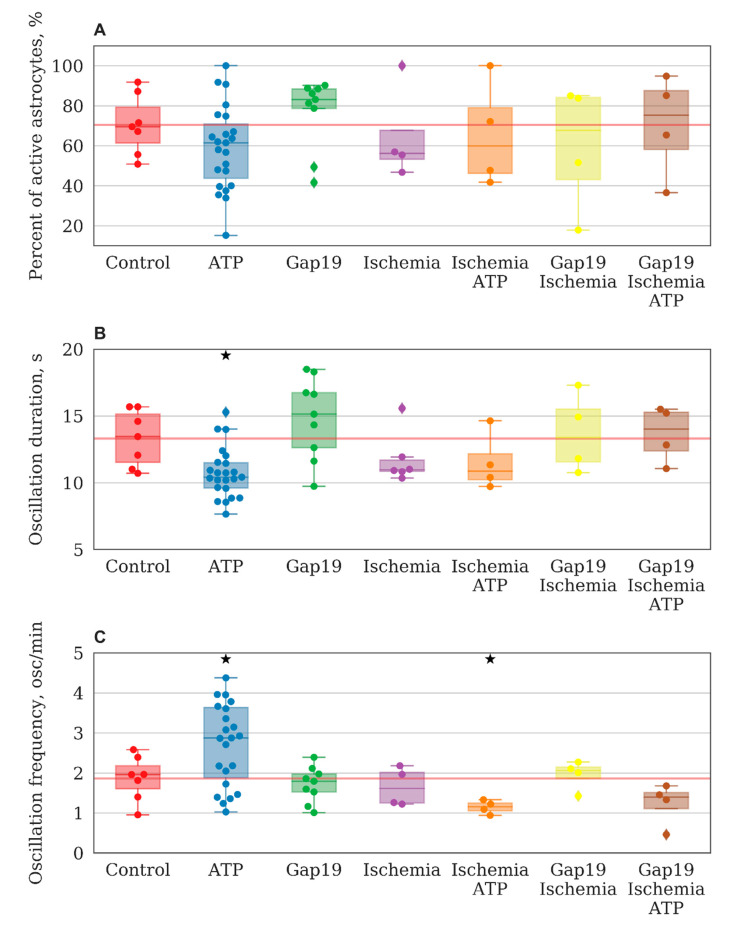
Main parameters of functional calcium activity in primary astrocyte cultures in the context of ATP and Gap19 influence under normal conditions and 7 days after ischemia-like condition modeling. (**A**) Proportion of astrocytes exhibiting calcium activity; (**B**) Duration of Ca2+ oscillations, s.; (**C**) Number of Ca2+ oscillations per min. ★—versus baseline (before application), *p* < 0.05, Wilcoxon rank-sum test.

Analysis of the main parameters of functional calcium activity in primary astrocyte cultures revealed that ATP addition did not cause significant changes in the percentage of cells that exhibited calcium activity. The percentage of oscillated cells in the “ATP” group was 61.47 [43.7; 70.9]% (“control” 69.5 [61.37; 79.33]%, Figure 3A). At the same time, significant changes in the frequency (before ATP addition 1.959 [1.605; 2.177] osc./min, after ATP addition 2.874 [1.884; 3.635] osc./min, Figure 3C) and duration (before ATP addition 13.449 [11.542; 15.135] s, after ATP addition 10.421 [9.611; 11.492] s) of calcium oscillations are shown (Figure 3B).

**Figure 4:**
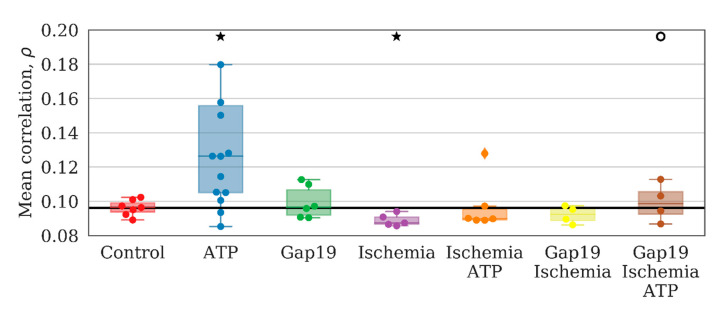
Mean value of the correlation maximum on the shift between pairs of average astrocyte calcium levels over time. ★—versus “Control”, ○—versus “Ischemia”, *p* < 0.05, the Kolmogorov–Smirnov test.

Studies on the characteristics of astrocyte connectivity showed that ATP application led to a significant increase in the mean values of the maximum correlation between pairs of average astrocyte calcium levels over time by 20% relative to the baseline (*p* < 0.05 the Kolmogorov–Smirnov test) (Figure 4). In the “ATP” group, an absolute change from 0.096 [0.094; 0.099] to 0.126 [0.105; 0.156] was identified. The application of Gap19 did not significantly change this parameter.

An analysis of network characteristics revealed that ATP application leads to a significant increase in the number of long-distant astrocytic connections that can be considered an emergence of complex interactions between astrocytes. The total number of connections per series increases from 732 [548; 796] to 2133 [515; 5134] (the Kolmogorov–Smirnov (KS) test). Such interactions cannot be considered a result of a simple increase in the frequency of calcium oscillations. At the same time, the number of correlated distant astrocytes was also increased.

**Figure 5:**
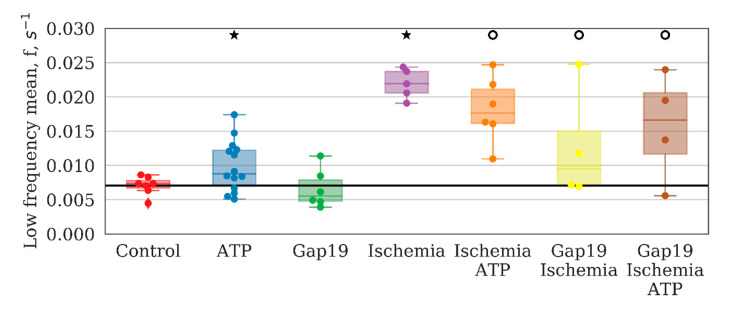
Average low-frequency component of calcium intensity in primary astrocyte cultures after ATP and Gap19 addition. ★ ATP—versus baseline, *p* < 0.05, t-criteria (*p* = 0.004); ★—versus “Control”, *p* < 0.05, the Kolmogorov–Smirnov test (*p* = 0.005); ○—versus “Ischemia”, *p* < 0.05, t-criteria (*p* = 0.004).

The most important parameters that characterize network activity are the distribution of calcium signals between astrocytes in a culture, including the time of signal propagation and frequency characteristics of calcium oscillations. The application of ATP increased the number of high-frequency events and suppressed low-frequency events (Figure 5). To characterize the frequency of calcium events, we computed the power spectral density of the cell calcium intensity time series, and took the frequencies corresponding to the 10th and 90th signal energy percentiles as the characteristic lower and upper frequency bounds. These bounds were then averaged over the whole image field.

These findings indicate changes in the activity profile of the entire system in monoastrocytic culture. The low-frequency events are most likely associated with long-term metabolic changes and are fairly stable under normal conditions.

**Figure 6:**
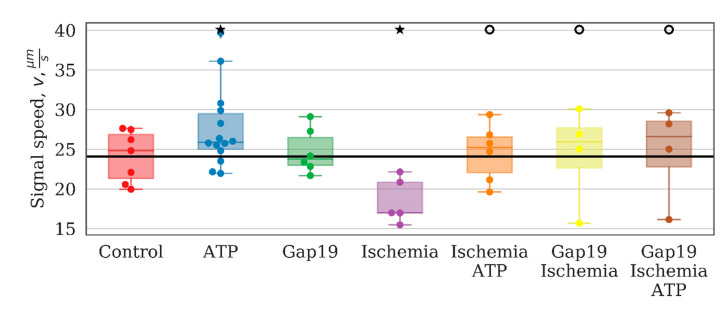
The rate of signal delay between astrocytes. ★ Gap19—versus baseline, *p* < 0.05, t-criteria (*p* = 0.036); versus “Control”, *p* < 0.05, the Kolmogorov–Smirnov test (*p* = 0.018). ○—versus “Ischemia”, *p* < 0.05, the Kolmogorov–Smirnov test (*p* = 0.006).

The study of the effects of connexin 43 blocker (Gap19) on the main parameters of network activity showed that despite the great physiological importance of this type of astrocytic connexin, significant changes were detected only in the speed of signal propagation between astrocytes under normal conditions (Figure 6). The percentage of cells that exhibited Ca2+ activity did not change significantly and was 83.1 [78.7; 88.4]% (Figure 3A). There were also no significant alterations in the duration (15.130 [12.634; 16.740] s) or frequency (1.792 [1.524; 1.975] osc./min) of Ca2+ events (Figure 3B,C).

An analysis of network connectivity parameters did not reveal significant changes in the network characteristics of the astrocytic network under the selective blocker Gap19 application. Therefore, the role of connexin 43 in the formation of correlated calcium dynamics in primary astrocyte cultures under normal conditions is not significant.

### 2.2. Correlated Astrocytic Response to Ischemic Influence

The next stage of our study aimed to analyze changes in the adaptive capacity of the astrocytic network in response to modeling ischemia-like conditions. Hypoxia is one of the most common experimental models of brain cell damage [18]. Several studies have shown a significant decrease in the viability and functional activity of brain cells in models of 10-min acute hypoxia in vitro [18,19,20]. However, astrocytes are considered more resistant to hypoxic damage than neurons. In this regard, to create stress conditions that affect astrocytic network functional activity, we adapted a model of oxygen-glucose deprivation (“ischemia”) lasting for 30 min (see the materials and methods). The results showed a significant decrease in the viability of primary astrocyte cultures in the “ischemia” group (see Supplementary Materials 1, Supplementary Materials Table S1), whereas blockage of Cx43 during ischemia modeling maintained the viability of astrocytes at the intact culture level (“control” 98.25 [96.99; 99.197]%, “ischemia” 95.59 [94.55; 97.35]%, “ischemia+Gap19” 99.21 [97.61; 100.0]%).

The number of cells that manifested calcium activity matched the control group and was 56.178 [53.258; 67.692]%. Furthermore, no statistically significant differences in the frequency and duration of Ca2+ oscillations were detected (1.612 [1.25; 2.01] osc./min and 10.967 [10.854;11.708] s, respectively) (Figure 3B,C).

Changes in the pattern of response to ATP stimulation in astrocytic cultures after ischemia-like condition modeling are of particular interest (see Supplementary Materials 1, Supplementary Materials Table S1). Under normal conditions, an increase in the frequency of Ca2+ oscillations after ATP application was observed. In contrast, the frequency of Ca2+ events in response to ATP application was decreased significantly in the “ischemia+ATP” group (1.156 [1.052;1.249] osc./min) (*p* < 0.05, Wilcoxon rank-sum test) (Figure 3C). The percentage of working cells and the duration of Ca2+ oscillations did not change. Therefore, ischemia leads to the loss of the functional response of astrocytes to the stimulating effect of ATP.

**Figure 7:**
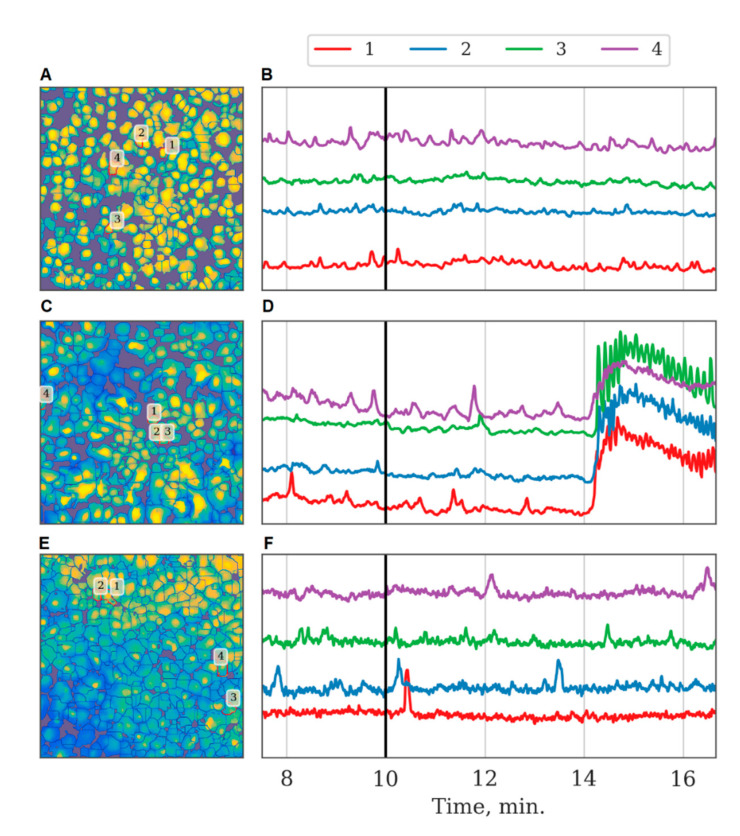
Characteristic examples of the dynamics of Oregon Green calcium sensor fluorescence in primary monoastrocytic cultures in vitro. (**A**,**B**)—normal conditions, PBS application; (**C**,**D**)—normal conditions, ATP applications; (**E**,**F**)—ischemic model, ATP application. (**A**,**C**,**E**)—examples of culture images segmented according to astrocyte positions. (**B**,**D**,**F**)—dynamics of calcium sensor fluorescence in selected astrocytes, note the correspondence between the astrocyte numbering and line colors. Vertical black lines indicate the moments of PBS or ATP application.

Analyzing astrocytic connectivity in the culture after ischemia modeling is of particular interest. While the primary astrocytic cultures in the post-ischemic period exhibited calcium events, their degree of synchrony and correlation was substantially different compared with that of the control cultures. In particular, the modeled ischemia-like conditions manifested as a significant decrease of all the parameters that characterized the connectivity of astrocytes. The number of direct long-distance connections was reduced by a factor of 30 (from 3.95 [3.1; 4.47] to 0.13 [0.07; 0.32]) (Figure 2). In the “ischemia” group, the average value of the maximum correlation of the shift between the average calcium level of neighboring astrocytes over time was 0.110 [0.107; 0.123], which is 2 times lower than that in the “control” group (0.242 [0.226; 0.263]; *p* < 0.05, the KS test). The number of long-distance connections in the “ischemia” group was 0.087 [0.087; 0.091] (“control” 0.096 [0.094; 0.099], *p* < 0.05, the KS test). Importantly, ischemia leads to changes in the astrocytic response to ATP stimulation in the post-ischemic period. Characteristic examples of changes in the dynamics of astrocytic calcium activity after ATP application in normal conditions and in model ischemia-like conditions are presented in Figure 7. Despite the statistically significant increase in distant connections after ATP application, similar to the control group (Figure 2), the average correlation level remained unchanged (Figure 4), which indicates the impairment of the functional astrocytic network.

The frequency characteristics, particularly the average low-frequency component of calcium intensity, of the astrocytic network were also changed significantly after ischemia-like condition modeling. The average frequency of base-level oscillations was increased by a factor of 3 relative to the control values on day 7 after ischemia influence and was 0.0219 [0.0206; 0.0237] (*p* < 0.05, the KS test). Moreover, the signal delay rate between astrocytes was reduced significantly (“control”: 24.81 [21.32; 26.85], “ischemia”: 16.98 [16.96; 20.84], *p* < 0.05, the KS test).

Investigation of the role of connexin 43 in the regulation of astrocytic resistivity to ischemic conditions revealed that the Cx43 blockade during ischemia modeling improves the viability of cells in the post-ischemic period. On the seventh day from the start, the number of viable cells in the “ischemia+Gap19” group was not distinguishable from that in the control group (Supplementary Materials 1).

Assessment of the functional calcium activity in the “ischemia+Gap19” group did not reveal significant changes in the main parameters of functional Ca2+ activity compared with the “ischemia” and “control” group values on day 7 after induction of the ischemia model (Figure 3).

In contrast, the application of Gap19 during ischemia modeling maintained all parameters of network connectivity. The average number of long-distance connections of an astrocyte, the average value of the correlation between pairs of astrocytes, average low-frequency component of calcium intensity, and the signal delay rate were preserved at the level of the control values. Furthermore, partial normalization of the response to ATP application was observed, namely, the increasing average correlation of astrocytic activity, although the number of distant connections of a single astrocyte and the frequency of calcium oscillations were not significantly different. Thus, the blockade of hemichannels during ischemic modeling preserves the connectivity of astrocytes in the post-hypoxic period.

## 3. Discussion

Earlier studies showed that astrocytes interact with each other by transferring low-molecular-weight compounds through gap junctions [17]. This process not only enables the long-distance transfer of energy substrates but is also important for the activation of several ATP-dependent reactions in two adjacent astrocytes. Calcium waves can propagate between astrocytes and form complex spatial-temporal patterns of Ca2+ activity [5]. However, whether astrocytes can regulate metabolic activity and autonomously provide a systematic response to stress conditions independently of neurons remains unclear.

The most important issue in investigating the collective actrocytic response is defining calcium activity characteristics that would reflect the network behavior. Our proposed approach to dynamic astrocytic network construction allows us to identify functional connections between astrocytes and determine the presence and changes of network activity. The developed algorithm represents the astrocytic network as an oriented graph, the vertices of which correspond to separate astrocytes, while the edges designate above-threshold correlations between astrocytic events. When the correlation between a pair of astrocytes is maximal for a significant time shift between calcium signals, it is attributed to the directional coupling. In turn, such causality allows us to define and estimate the rate of calcium wave propagation in primary astrocyte culture.

Selecting a threshold for the correlation value is crucial for identifying and tracking astrocytic networks. The choice is not apparent since even statistically independent random time series can yield nonzero correlations over finite time windows. Moreover, the characteristic level of spurious correlations would not be universal and depends on particular signals. To circumvent the problem, we defined the ‘baseline’ correlation level as the correlation value for the most distant astrocytes in the observation field in the control case, assuming that little or no actual interaction exists between them. The threshold chosen in this way is equally applicable to astrocyte cultures subject to external stimuli. In this case, changes in the network statistics provide insight into the modification of the astrocytic interaction network.

The developed algorithms are integrated into the custom-written software package, which performs complete analysis and data processing from the obtained microscope images to the dynamic network construction and can be a convenient tool for analyzing image series of cell metabolic activity. The approach currently applied to study the behavior of astrocytic cultures can further be used to analyze network activity in neuronal cultures and mixed neuron-glial cultures. More generally, the developed algorithms allow the processing of series of images obtained from arbitrary cell cultures with some intracellular chemical dynamics.

Our studies revealed that astrocytes exhibit comparatively low network activity under normal conditions in vitro. The number of long-distance connections and the level of correlations increase significantly in response to an increase in intercellular ATP concentration, which cannot be explained by simple ATP-mediated activation of calcium homeostasis, as the calculated correlations are insensitive to the changes in the base level of calcium activity and the frequency of calcium oscillations. This demonstrates that astrocytes can become organized into a functional dynamic network in response to external stimulation.

Ischemia modeling has a significant impact on network activity, manifested by the decrease in all values that characterize network connectivity. The results also highlight important changes in astrocyte responses to ATP stimulation after ischemic influence. We hypothesize that the depression of the ATP-stimulated network emergence in this case is due to adaptive astrocytic reactions. Several studies addressed the changes in the functional activity of neuronal networks in hypoxic and ischemic lesions. Earlier, we demonstrated that hypoxia leads to the dramatic suppression of network interactions between neural cells, up to complete disruption, that is manifested in the alterations of both the calcium and bioelectric activity, as recorded by multi-electrode matrices [19,20]. At the same time, little is known regarding the network activity of mono-astrocytic cultures. Here, we are the first to show that while the calcium oscillations persist in the post-ischemic period, the correlations between cells are almost completely lost. Importantly, 10 µM ATP stimulation in normal conditions increases the frequency and improves correlations between calcium oscillations in distant astrocytes, such that a large-scale connected functional network emerges. In the post-ischemic period, such a response to ATP is absent, which suggests deep functional impairment of the astrocytic network.

There are experimental data showing changes in gliotransmitter regulation after hypoxic influence. It has been established that ATP release in the brain stem, presumably carried out by astrocytes, helps maintain cellular respiration and counteract hypoxia. It has been demonstrated that acute systemic hypoxia (a 5-min reduction in oxygen concentration in the breathable air to 10%) causes the release of a key gliotransmitter (ATP) in the brain stem areas responsible for the formation of breathing rhythm. Blockade of ATP receptors in the same brain stem area alleviates hypoxia-induced respiratory depression [21].

Interestingly, blockade of the main type of astrocytic connexins Cx43, which should provide a metabolically consolidated response under stress, preserves network parameters in primary astrocyte cultures for at least 7 days after the modeled ischemia-like conditions. A substantial increase in cell viability in primary astrocyte cultures in ischemia modeling and blockade of gap junctions was also observed. Accumulated experimental data indicate that Cx43 expression in astrocytes increases under hypoxic influence, and Cx43 plays an important role in cell death and neuronal damage caused by cerebral ischemia [22,23]. It is assumed that ischemia/reperfusion leads to an increase in the concentration of extracellular Ca2+ ions, the release of inflammatory factors, and, as a result, the activation of astrocytic hemichannels [24]. Hemichannels consisting of Cx43 can also be activated by kinase p38 and proinflammatory cytokines released by activated microglia [25]. This leads to an uncontrolled release of ATP, glutamate, and calcium, leading to calcium overload and tissue excitotoxicity [8]. Our data on the preservation of cell viability and network parameters of astrocytic cultures in the remote posthypoxic period are consistent with a study demonstrating that the use of the Cx43 blocker Gap26 improves neurological function in animals and decreases the incidence of heart attack in an ischemia model in vivo [26]. Therefore, Cx43 could be a promising therapeutic target in the development of methods for brain ischemia protection. This is an exciting area for upcoming research.

The current study revealed the emergent property of astrocytes to functionally unite in a large-scale network, and the Cx43 inhibition in ischemic conditions may be considered a favorable factor. In this regard, it is essential to further investigate astrocytic interactions as a factor in brain adaptation to ischemic damage, especially changes in these adaptive properties under physiological astrogliosis.

## 4. Materials and Methods

### 4.1. Ethics Statement

All experimental procedures were approved by the Bioethics Committee of Lobachevsky University and carried out in accordance with Act 708n (23 082010) of the Russian Federation National Ministry of Public Health, which states the rules of laboratory practice for the care and use of laboratory animals, and Council Directive 2010/63 EU of the European Parliament (22 September 2010) on the protection of animals used for scientific purposes. Newborn C57BL/6 (P1-P3) mice were killed by cervical vertebra dislocation.

### 4.2. Isolation of Primary Astrocyte Cultures

Primary astrocyte cultures were obtained from the cerebral cortex of newborn C57BL/6 mice (1–3 days after birth). The use of brain tissue in the early postnatal period minimizes the risk of the presence of nondifferentiated astrocytes in cultures.

Preparation and long-term culture of primary astrocyte cultures were performed in accordance with a protocol based on studies by [27] and [28] with several modifications. Surgically isolated cerebral cortices were cleared from the meninges and then mechanically dissected. To disrupt interconnections between cells, the tissue was additionally incubated with 0.25% trypsin solution (Thermo Fisher Scientific, Waltham, MA, USA) for 20 min in a CO_2_ incubator (BINDER GmbH, Tuttlingen, Germany). Next, the cell suspension was washed three times in phosphate-buffered saline (PBS) and once in Dulbecco’s Modified Eagle Medium (DMEM) containing 4.5 g/L glucose and supplemented with 0.5% L-glutamine, 1% B27 (Thermo Fisher, Waltham, MA, USA), 0.1% sodium pyruvate (Thermo Fisher, Waltham, MA, USA), and 10% fetal bovine serum (PanEco, Moscow, Russia). The suspension of dissociated cells was centrifuged at 800 rpm for 3 min. Then, the pellet was resuspended in culture medium, and, the obtained suspension was placed on coverslips pretreated with polyethylenimine solution (1 µg/mL) (Merck KGaA, Darmstadt, Germany), which provides effective cell attachment to the substrate. The initial cell density was 4500 cells/mm2. Primary astrocyte culture viability was maintained under constant conditions of 35.5 °C, 5% CO_2_, and a humidified atmosphere in a cell culture incubator for more than 30 days. Half of the medium was replaced every third day.

### 4.3. Immunocytochemical Analysis

To verify the cellular content, primary astrocyte cultures were subjected to immunocytochemical staining on day 21 of culture development in vitro (DIV) (Supplementary Materials 1, Supplementary Materials Figure S1). The cultures were fixed with 4% paraformaldehyde for 15 min at room temperature, followed by incubation with a solution of 0.2% Triton X-100/PBS for effective cell permeabilization. For immunofluorescence reactions, the cultures were then incubated for 2 h in the presence of a polyclonal chicken anti-GFAP (glial fibrillary acidic protein, marker of differentiated astrocytes) primary antibody (Abcam, Cambridge, UK, 1:1000 dilution) and polyclonal goat anti βIII-tubulin (marker of differentiated neurons) primary antibody (Abcam, Cambridge, UK, 1:750 dilution). Next, the cultures were subjected to a 45-min incubation in the following secondary antibody mixture: mouse anti-chicken Alexa 647 (Thermo Fisher Scientific, Waltham, MA, USA, 1:100 dilution) and rabbit anti-mouse Alexa Fluor 555 (Thermo Fisher Scientific, Waltham, MA, USA, 1:100 dilution). The stained material was observed using a Zeiss 510 NLO fluorescence confocal microscope (Carl Zeiss, Oberkochen, Germany).

### 4.4. Calcium Imaging

The functional calcium activity of astrocytes was studied using an LSM 510 laser scanning microscope (Carl Zeiss, Oberkochen, Germany) with a W Plan-Apochromat 20×/1.0 objective. The calcium imaging technique allowed visualization of the functional architecture of cells in culture. We used the fluorescent calcium-sensitive dye Oregon Green 488 BAPTA-1 AM (OGB-1) (0.4 μM, Thermo Fisher Scientific, Waltham, MA, USA) dissolved in dimethylsulfoxide (DMSO) (Merck KGaA, Darmstadt, Germany) with 4% Pluronic F-127 (Thermo Fisher Scientific, Waltham, MA, USA). OGB-1 was added to the culture medium and incubated for 40 min in a CO2 incubator. The fluorescence of OGB1 was excited at 488 nm by argon laser radiation, and emission was recorded in the range of 500 to 530 nm. The dynamics of intracellular calcium concentration were measured by analysis of a time series of 512 × 512 pixel images capturing 420 μm × 420 μm fields of view that was recorded at 2 Hz. The following parameters of the functional calcium activity were assessed: duration of the calcium oscillations (time from the beginning to the end of an oscillation (s)), frequency of calcium oscillations (average number of oscillations per min), and percentage of working cells (ratio of the number of cells in which at least one oscillation was recorded among the total number of cells (%)) [29,30].

### 4.5. Ischemia-Like Condition Model

Ischemia-like conditions were modeled on day 21 of primary astrocyte culture development in vitro by replacing the culture medium with a medium with low oxygen (0.37 mL/L) free of glucose, lactate, and pyruvate for 30 min. The experiment was performed in a sealed chamber in which air was also replaced by argon gas.

### 4.6. Cell Viability Analysis

To identify the nuclei of dead cells and the total number of cell nuclei in the primary astrocyte cultures, propidium iodide (Merck KGaA, Darmstadt, Germany) and bis-benzimide (Merck KGaA, Darmstadt, Germany) were used. Solutions of 5 μg/mL propidium iodide and 1 μg/mL bis-benzimide were added separately to the culture medium 30 min before viability registration. The stained cultures were observed using a ZEISS Observer A1 inverted fluorescence microscope (Carl Zeiss, Oberkochen, Germany). The proportion of dead cells was calculated as the ratio of nuclei stained with propidium iodide to the total number of nuclei.

### 4.7. Biochemical Screening

To analyze the features of the functional activity of astrocytes, the following agents were added to the culture medium on day 28 of culture development in vitro: ATP (10 µM, Merck KGaA, Darmstadt, Germany) as an activator of calcium activity and Gap19 (100 µM, Merck KGaA, Darmstadt, Germany) to estimate the role of connexin blockade in the implementation of functional calcium reactions. The same volume of PBS was used as a control. The baseline activity was recorded for 10 min.

### 4.8. Calcium Event Analysis

Calcium event detection in astrocytes was performed by a previously developed algorithm [16]. Input data for the algorithm consist of two image series with the same dimensionality that are obtained from the same microscope: the astrocyte activity and images of a cell-free medium. The software pipeline includes the following steps:

1. To correctly measure relative changes of a signal, we subtract a constant offset, given by the time average of the cell-free image series.
2. In the second step, the two filters are applied sequentially: VBM3D signal filtering and spatial-temporal 3D box filtering.
3. Based on the cell-free image series, we evaluate the baseline of the calcium activity for each pixel. Next, the relative signal changes are estimated by referring to the baseline.
4. The astrocytic dynamic activity is defined as the signal that exceeds a spatial adaptive threshold above the baseline level. The adaptive threshold is computed by the statistical model of noise reduction adjusted according to the cell-free images (see details in [16]).
5. In the last step of the algorithm, spatiotemporal clustering of activity patterns into calcium events is performed by DBSCAN over time and by a window-based approach over space.

Initially, the entire image plane is considered, and events are identified as spatiotemporally connected areas with significant activity. Next, the watershed method is applied to the long-exposure calcium intensity image to discriminate the areas corresponding to individual astrocytes. Finally, calcium events are segmented according to astrocyte regions. Further analysis utilizes the obtained individual astrocyte calcium events and filtered intensity signals.

### 4.9. Construction of a Dynamic Astrocytic Network

The approach for reconstruction of an astrocytic network is based on calculating the Pearson correlation coefficient between filtered signals of each cell pair:

(1) $$\rho_{ij}=\frac{\sum_{k=1}^{n}{\overset{ˇ}{x}}_{k}^{i}\;{\overset{ˇ}{x}}_{k}^{j}}{\sqrt{\sum_{k=1}^{n}{\left({\overset{ˇ}{x}}_{k}^{i}\right)}^{2}\sum_{k=1}^{n}{\left({\overset{ˇ}{x}}_{k}^{j}\right)}^{2}}},$$

(2) $${\overset{ˇ}{x}}_{k}^{i}=x_{k}^{i}-{\langle x_{s}^{i}\rangle }_{k-w,k}$$

$x_{k}^{i}$—calcium signal of i-th cell at time k,
${\overset{ˇ}{x}}_{k}^{i}$—calcium signal minus moving average with window size w,
${\langle x_{s}^{i}\rangle }_{k-w,k}$—average of signal in range [k−w,k].

The astrocytic network is represented as an undirected graph, where vertices correspond to cells, and edges are drawn between the cells for which the correlation coefficient exceeds a certain threshold. Cellular signals are characterized by two main quantities: the level of intracellular calcium and the size of a calcium event inside the cell. Both are used to construct the network. Furthermore, the propagation of calcium signals between cells leads to detectable time delays in calcium elevations (Supplementary Material Movie 1) and in certain cases allows for assignment of a directed edge. Different time delays were probed to choose the one for which the correlation between a pair of cells would be maximal (Supplementary Material 2).

A threshold was set to reject spurious correlations between cell calcium signals that would be caused by coincidence rather than actual interaction. The choice of the threshold was based on the following. Since the direct interaction between astrocytes is local, the mutual influence should decrease with distance. Therefore, the baseline level of correlations can be estimated from values that are found for remote astrocytes. Given the typical size of an astrocyte up to 40 µm, we referred to the correlation level between cells separated by at least 100 µm as the baseline [31].

**Figure 8:**
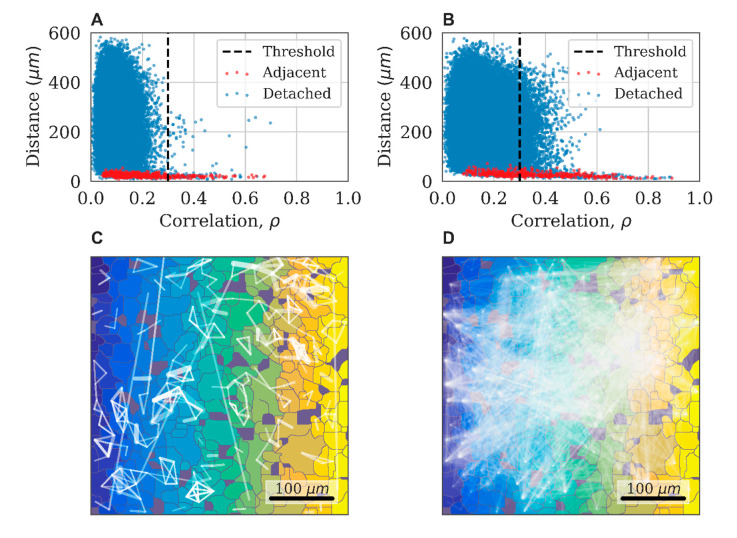
The distance–correlation relationship between pairs of neighboring (red) and distant (blue) astrocytes in the control state (**A**) and after applying ATP; (**B**) An example of a correlation network of astrocytes with a threshold ρ>0.3 for the control state (**C**) and after applying ATP (**D**)**.**

The implementation of this approach is illustrated in Figure 8A,B, which shows a typical relationship between the level of correlation and the distance in pairs of astrocytes in the control experiment. Adjacent cells are marked in red. Three indicative groups of points are highlighted in the figure. For the nearby and directly interacting astrocytes (distance between central points < 40 µm), the correlation coefficient can reach 0.9. For distant astrocytes (distance > 300 µm), the correlation does not exceed 0.3. We used this value as a threshold to distinguish a significant correlation between pairs of astrocytes. The third group of points is represented by pairs of astrocytes located at distances ranging from 40 to 300 µm with cross-correlation values exceeding 0.3. These properties are interpreted as the result of an indirect dynamic interaction between astrocytes and almost do not occur in the control group.

The correlation astrocytic network is constructed as follows: the vertices of the graph are mapped to astrocytes, and the presence of a significant level of correlation between pairs of astrocytes (correlation greater than 0.3) is indicated by an edge connecting the corresponding vertices. A characteristic example of the obtained network for the control group is presented in Figure 8C. Such networks typically display a sufficiently large number of small local groups.

### 4.10. Dynamic Astrocytic Network Analysis

The impact of external factors on astrocytic culture can lead to changes in the correlation and dynamic properties of the system. As a result of exposure to ATP, the point cloud expands and shows higher correlation values at long distances (Figure 8A,B). This reflects an increase in the connectivity of the dynamical astrocytic network. While the original graph would contain several disconnected subgraphs, the resulting correlation astrocytic network manifests the so-called giant component, a connected subgraph of the size of the order of the entire network, due to the emerging large number of long-range functional connections (Figure 8D).

Network analysis focuses on the following key features: the number of functional connections between astrocyte pairs, the average number of connections between astrocytes, the average propagation speed of delays between signals, the average correlation level of network cells, and the frequency of cell signals, as described in detail in Supplementary Material 2.

### 4.11. Statistical Analysis

The astrocytic responses to various biochemical stimuli were investigated using statistical analysis. Astrocyte cultures were divided into different groups. The control group was designated as representing a normal state of astrocytic activity.

The influence of external factors on the astrocyte state was determined by comparing feature samples of the control and case groups by the one-sided two-sample Kolmogorov–Smirnov test. This is a nonparametric test that quantifies the distance between the empirical distribution functions of two samples [32]. The computed features of some groups violate the normality assumption for parametric tests. The normality of distributions is tested by applying the Kolmogorov–Smirnov test (KS test) (*p* < 0.05 for some groups, but not for all). To overcome this, a two-sample KS test was applied. Determination of the distribution shift direction was provided by a one-sided KS test. To assess whether group mean ranks differ, the Wilcoxon rank-sum test was applied to the data. A t-test was performed to compare group values with the initial baseline.

Statistical significance was determined using stats module of SciPy library [33]. The one-sided two-sample KS test is performed by the ks_2samp function. The Wilcoxon rank-sum test is performed by the Mann-Whitney function. Differences between groups were considered statistically significant if *p* < 0.05.

Descriptive statistics of each feature per group are represented as “M [Q1; Q3]”, where M—median, Q1—first quartile (quantile 0.25), and Q3—third quartile (quantile 0.75) of the group samples.

## 5. Conclusions

We developed a novel mathematical approach to reconstruct functional astrocytic networks based on an analysis of dynamic correlations between calcium events. This approach revealed dramatic changes in the structure and properties of astrocytic networks in normal and ischemia-like conditions.

## Supplementary Materials

The following are available online at https://www.mdpi.com/1422-0067/21/21/7952/s1, Figure S1: Morphology of primary astrocyte cultures on day 21 of cultivation in vitro, Table S1: Cell viability analysis of primary astrocyte cultures on day 7 after modeled stress factors, Video S1: the propagation of calcium signals between astrocytes.
