# Supplementary material for: Signatures of the Consolidated Response of Astrocytes to Ischemic Factors In Vitro
Source: Int J Mol Sci. 2020 Oct 26;21(21):7952. doi: 10.3390/ijms21217952 (PMC7672566; doi:10.3390/ijms21217952)
Supplement: Supplementary file 1 [file ijms-21-07952-s001.zip › Supplementary_material_2.docx]

Supplementary Material 2

***Construction of a dynamic astrocytic network***

The dynamic neural-astrocytic network is presented as an undirected graph $G=(V,E)$, where vertices $V$ correspond to cells, and edges $E$ indicate correlations between cells, the value of which exceeds a specific threshold:

$$V=\bar{1, n}, E=\left\{ (i,j):i,j\in V, \rho_{ij}>\rho_{thr} \right\},$$

where $\rho_{ij}$ *–* Pearson correlation coefficient between two cells $i, j\in V$*:*

$$\rho_{ij}=\frac{\sum_{k=1}^{n} \check{x}_{k}^{i} \check{x}_{k}^{j}}{\sqrt{\sum_{k=1}^{n} \left( \check{x}_{k}^{i} \right)^{2}\sum_{k=1}^{n} \left( \check{x}_{k}^{j} \right)^{2}}},$$

$$\check{x}_{k}^{i}=x_{k}^{i}-\left\langle x_{s}^{i} \right\rangle_{k-w,k}$$

$x_{k}^{i}$ *–* calcium signal of $i$-th cell at time $k$*,*

$\check{x}_{k}^{i}$ *–* calcium signal minus moving average with window size $w$*,*

$\left\langle x_{s}^{i} \right\rangle_{k-w,k}$ *–* average of signal in range $[k-w,k]$*.*

The calcium signal of a cell can be characterized by two quantities: the level of intracellular calcium $C_{k}^{i}$ and event size $A_{k}^{i}$, which is numerically equal to the number of active pixels. Both can be used to calculate the correlation coefficient between cell pairs. First, signals are transformed by moving average subtraction to eliminate general signal trends. Then, the Pearson correlation coefficient is estimated between cell couples.

The propagation of calcium signals between cells can lead to time delays and make graphs directed. Therefore, we search for the maximal correlation between each pair of cells, attempting different time shifts in the range of $10$ frames ($W$). The edge is set if the correlation between time-shifted signals is above a certain threshold, and its direction is chosen such that the corresponding time delay is positive.

$$V=\bar{1, n}, E=\left\{ (i,j):i,j\in V, \tilde{\rho}_{ij}>\rho_{thr} \right\}$$

$$\rho_{ij}\left( \tau\right)=\frac{\sum_{k=\min\left( -\tau, 1 \right)}^{n-\max\left( \tau,0 \right)} \check{x}_{k}^{i} \check{x}_{k+\tau}^{j}}{\sqrt{\sum_{k=1}^{n} \left( \check{x}_{k}^{i} \right)^{2}\sum_{k=1}^{n} \left( \check{x}_{k}^{j} \right)^{2}}},$$

$$\tilde{\rho}_{ij}=\rho_{ij}\left( t \right),$$

$$\tau_{ij}=\mathrm{argmax} \rho_{ij}(\tau).$$

where $\rho_{ij}\left( \tau\right)$ – correlation coefficient between signals with shift $\tau$,

$\tilde{\rho}_{ij}\left( \tau\right)$ – maximal correlation coefficient over various shifts in the range $[-W;W]$,

$\tau_{ij}$ – time delay at the maximum correlation level.

***Dynamical astrocytic network analysis***

An assessment of the influence of external factors on astrocyte culture dynamic properties of the system is performed. Below is a detailed description of network analysis considering the following key features: the number of functional connections between astrocyte pairs, the average number of connections between astrocytes, the average propagation speed of delays between signals, the average correlation level of network cells and the frequency of cell signals.

1. the number of connections between astrocyte pairs

$$N_{c}=\left| E \right|;$$

The number of correlations between cells indicates the level of coherence of calcium events occurring in individual cells. An increase in the number of connections between astrocytes reflects the growth of coordination of cell dynamics. An absence of connections indicates the lack of synchronized pairs of astrocytes. Rare mutual connections show that part of the cells manifests coordinated activity, but most of the cells oscillate independently. Correlated dynamic clusters expand with an increasing number of links. Ultimately, a single cluster appears, and the level of mutual synchronization saturates.

1. the average number of connections between astrocytes

$$N_{n}=\left\langle\left| \left\{ j:j\in V, (i,j)\in E \right\} \right| \right\rangle_{i};$$

The average number of correlation connections per cell reflects the average number of cells synchronized with a reference cell.

1. the average propagation speed of delays between signals

$$S=\left\langle\frac{d_{ij}}{\tau_{ij}} \right\rangle_{ij}$$

where $d_{ij}$ *–* distance between centers of $i$-th and $j$-th cell*s,* $\tau_{ij}$ – time signal delay.

The delay $\tau_{ij}$ in the propagation of signals between cells is the time during which information about the calcium impulse in the first astrocyte reaches the second astrocyte, and it repeats the activity of the first astrocyte. Given the time $\tau_{ij}$ and distance $d_{ij}$, one can estimate the speed of information propagation between astrocytes. A high-impulse propagation speed between distant cells indicates active network interaction during signal transmission between neighboring cells.

1. the average correlation level of network cells

$$P=\left\langle\rho_{ij} \right\rangle_{ij};$$

This value has the properties of a correlation coefficient: a correlation level equal to $1$ indicates completely identical calcium signals in all cells. A level less than one indicates a certain level of synchronization. The significance of the correlation level is determined as an exceeding level in a certain undisturbed state of the astrocytic network.

1. the average network correlation level of adjacent cells

$$P_{a}=\left\langle\rho_{ij} \right\rangle_{ij}, \left( i,j \right)\in E_{p},$$

where $E_{p}$ – set of pairs of adjacent cells in space.

Signal transmission between cells occurs mainly between adjacent cells because astrocytes are organized into a local structure. The average correlation level of the entire network identifies the interaction of both far and near astrocytes. To characterize the interaction of space-adjacent astrocytes, one can consider the average level of correlation between adjacent astrocytes. More specifically, it is described as adjacent regions of the image that correspond to individual astrocytes.

1. the lower frequency of cell signals $\left\langle\omega_{l}^{i} \right\rangle_{i}$:

$$\frac{\int_{\omega_{l}}^{\omega_{h}} \left| \mathcal{F}\left[ \check{x}\left( t \right) \right]_{\omega} \right|d\omega}{\int_{-\infty}^{+\infty} \left| \mathcal{F}\left[ \check{x}\left( t \right) \right]_{\omega} \right|d\omega}>0.85,$$

where ℱ ***–*** Fourier transform symbol*.*

The major frequency domain is defined as the range between the points where the integrated power spectral density crosses $7.5\%$ and $92.5\%$ of the total power in the spectrum. The boundaries of the occupied bandwidth correspond to the lower $\left\langle\omega_{l}^{i} \right\rangle_{i}$ and upper $\left\langle\omega_{h}^{i} \right\rangle_{i}$ signal frequencies.

Estimation of the lower average frequency $\left\langle\omega_{l}^{i} \right\rangle_{i}$ of the signals of all cells shows the low-frequency component of the cell ensemble.
