# Supplementary material for: Signatures of the Consolidated Response of Astrocytes to Ischemic Factors In Vitro
Source: Int J Mol Sci. 2020 Oct 26;21(21):7952. doi: 10.3390/ijms21217952 (PMC7672566; doi:10.3390/ijms21217952)
Supplement: Supplementary file 1 [file ijms-21-07952-s001.zip › Supplementary_material_1.docx]

Supplementary Material 1

| 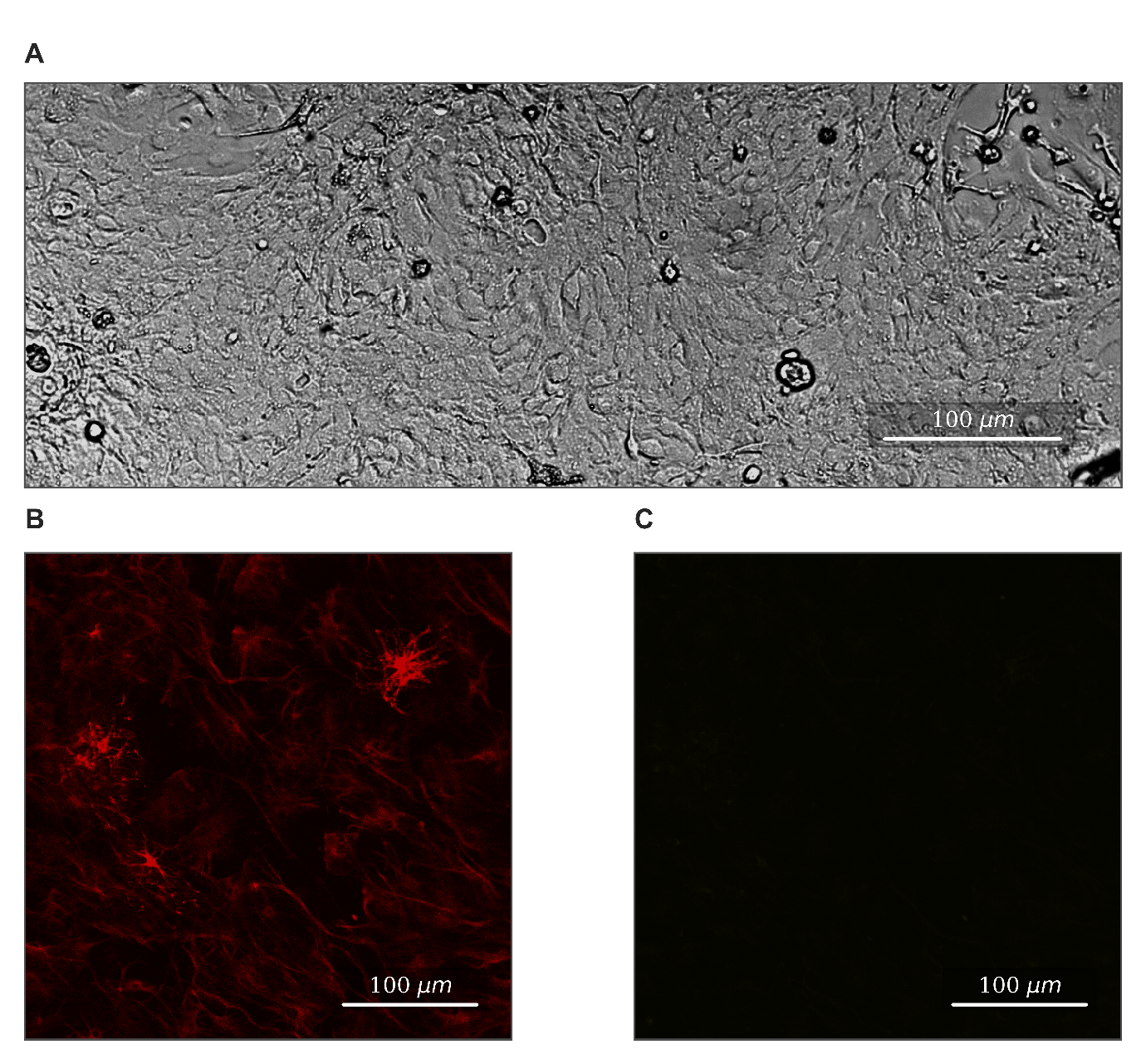 |  |
| --- | --- |

**Figure S1**. Morphology of primary astrocyte cultures on day 21 of cultivation in vitro. A – representative wide-field light microscopy image. B-C – immunocytochemical staining: B – representative confocal image of primary astrocyte culture stained with a marker of cytoskeleton protein of differentiated astrocytes (GFAP), C - representative confocal image of primary astrocyte culture stained by a marker of neuronal protein (βIII tubulin). Scale bars – 100 µm.

**Table S1.** Cell viability analysis of primary astrocyte cultures on day 7 after modeled stress factors

| **Group** | **Number of viable cells, %** |
| --- | --- |
| Control | 98.25 [96.99; 99.197] |
| Ischemia-like conditions | 95.59 [94.55; 97.35]* |
| Ischemia-like conditions + GAP19 | 99.21 [97.61; 100.0] |

* - versus “Control”, p < 0.05, the Kolmogorov–Smirnov test
